# Association between night work and dyslipidemia in South Korean men and women: a cross-sectional study

**DOI:** 10.1186/s12944-019-1020-9

**Published:** 2019-03-28

**Authors:** Jae Hong Joo, Doo Woong Lee, Dong-Woo Choi, Eun-Cheol Park

**Affiliations:** 1 Department of Public Health, Graduate School, Yonsei University, Seoul, Republic of Korea; 2 Institute of Health Services Research, Yonsei University, Seoul, Republic of Korea; 3 Department of Preventive Medicine, Yonsei University College of Medicine, 50 Yonsei-ro, Seodaemun-gu, Seoul, 03722 Republic of Korea

**Keywords:** Dyslipidemia, Night work, Eating habit, Sleep duration, White-collar

## Abstract

**Background:**

Previous studies have reported that an irregular work schedule, particularly nighttime work, is associated with an altered lipid profile. Additionally, a mismatch in circadian rhythm can affect sleeping and eating habits, leading to poor health. This study aimed to examine the association between night work and dyslipidemia among South Korean adults aged ≥30 years.

**Methods:**

For this study, the data of 5813 participants in the 2013–2016 Korea National Health and Nutrition Examination Survey were analyzed. Diagnoses of dyslipidemia were based on blood sampling tests of total cholesterol, high-density lipoprotein (HDL), low-density lipoprotein (LDL) cholesterol, and triglyceride levels. Night work was defined as that conducted during evening (6 P.M.–12 A.M.) and overnight hours (12 A.M.–8 A.M.). The association between night work hours and dyslipidemia in South Korean men and women was investigated using a logistic regression analysis.

**Results:**

After adjusting for sociodemographic, economic, health-related, and nutritional factors, an association of night work with dyslipidemia was observed in male participants (odds ratio = 1.53, 95% confidence interval: 1.05–2.24). In subset analyses of male participants, night workers who skipped meals were more likely to have dyslipidemia than their day-working counterparts. Among men who slept < 7 h, night workers had a higher probability of dyslipidemia than day workers. In contrast, no statistically significant association between night work and dyslipidemia was observed in female participants, although the probability of dyslipidemia appeared to increase with advancing age. Furthermore, when women with dyslipidemia were subdivided by occupational categories, night workers in white collar positions were more likely to have dyslipidemia than their day-working counterparts.

**Conclusion:**

Our study observed an association of night work with dyslipidemia, particularly in men. Although these findings may support interventions for South Korean night workers, further studies are needed for validation.

## Background

The concept of shift work arose from industrial growth and the increase of 24-h workplaces, which required continuous staffing and irregular work schedules [1, 2]. In South Korea, the prevalence of night and shift work is highest in the field of manufacturing, followed by wholesale and retail businesses [3]. Although no consensus has been reached regarding the definition of shift work, this term is often used in reference to work hours outside of the conventional daytime period.

The major difficulties associated with shift work mainly involve work conducted during evening or overnight hours, due to its effects on circadian rhythm. Changes in circadian rhythms can disrupt homeostasis and lead to the desynchronization of enzymatic activity and metabolic function [4]. For example, evidence suggests a correlation between an altered distribution of food intake due to a mismatch in circadian rhythm (e.g., nighttime food ingestion) and increased cholesterol levels [5]. Circadian rhythm disturbances have also been identified as a significant factor related to cardiovascular disease (CVD). For example, an inability of the circadian rhythm governing oxygen supply to adapt promptly to the changing demands of night work will likely lead to myocardial infarction [4]. Furthermore, night workers are more likely to experience fatigue due to a lack of sleep [6]. Although this relationship is poorly understood, sleep deprivation has been identified as a potential risk factor for CVD [7].

CVD is the cause of substantial societal burdens worldwide and is the leading cause of death in South Korea, where the CVD-associated mortality rate has been increasing gradually in recent years. In 2017, diseases of the circulatory system accounted for 21.5% of all deaths in South Korea, second only to neoplasms (28.1%) [8]. The prevalence of dyslipidemia, a major risk factor for CVD [9], is also increasing in South Korea [10], with reported rates ranging from 30 to 60% [10]. Although age, hypertension, and obesity are commonly known risk factors for dyslipidemia, these factors are better controlled and moderated today than in previous periods [11]. Therefore, the increased prevalence of dyslipidemia in South Korea is likely attributable to lifestyle factors.

Previous studies have reported associations between irregular work schedules, particularly night work, and altered lipid profiles [12, 13]. Therefore, preventive measures are needed to mitigate lipid disorders and ensure the well-being of workers during non-standard working hours. Night work appears to serve as barrier to a healthy lifestyle and a threat to well-being, as a circadian rhythm mismatch can disrupt adequate sleeping and eating habits, leading to poor health [14]. We hypothesize that in night workers, insufficient amounts of sleep and irregular eating habits may contribute to the onset of dyslipidemia. In this study, therefore, we aimed to investigate and elucidate the association of dyslipidemia with night work.

## Methods

### Study participants

**Fig. 1:**
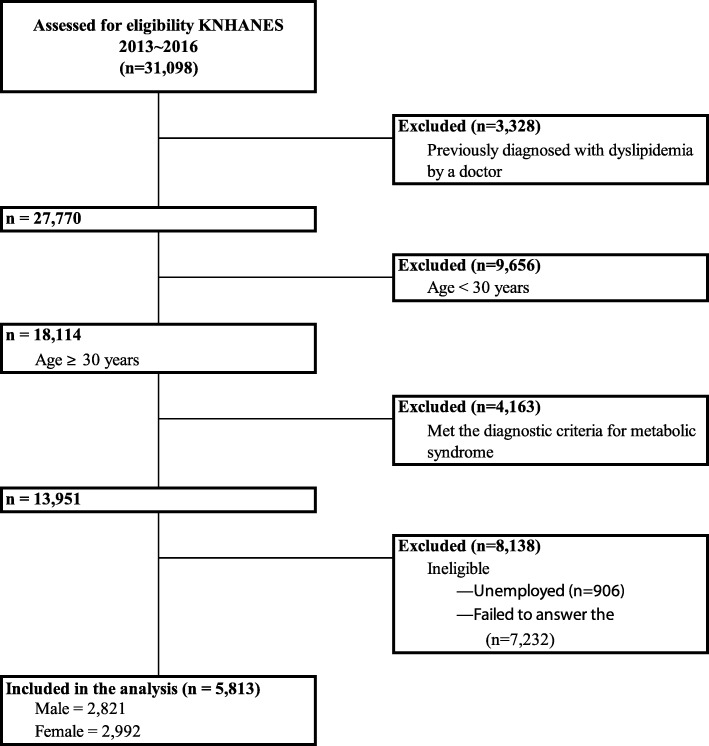
Flow diagram of subject inclusion and exclusion

We collected data from the 2013 to 2016 Korea National Health and Nutrition Examination Survey (KNHANES), which was conducted by the Korea Centers for Disease Control and Prevention (KCDCP). The KNHANES is a self-reported, nationally representative survey of South Koreans of all ages and is designed to gather annual national data on sociodemographic, economic, and health-related conditions and behaviors. Since 2007, the collected data have been subjected to an annual review and approval by the KCDCP Research Ethics Review Committee. The KNHANES 2013–2016 included 31,908 participants. We excluded 25,285 of these participants for various reasons (Fig. 1). First, participants with a previous clinical diagnosis of dyslipidemia were excluded, as this may have influence the reliability of the outcome (*n* = 3328). Second, in this study, dyslipidemia was diagnosed via a blood samples collected during the KNHANES. Therefore, people younger than 30 years were excluded because they did not undergo blood testing as part of the survey (*n* = 9656). Third, our study aimed to examine specific relationships with dyslipidemia. As dyslipidemia and metabolic syndrome share a few diagnostic components, including high-density lipoprotein (HDL) cholesterol and triglycerides levels, participants who met the modified National Cholesterol Education Program Expert Panel Adult Treatment Panel III (NCEP-ATP III) diagnostic criteria for metabolic syndrome with a lower waist circumference were excluded to increase the validity of our study (*n* = 4163) [15, 16]. Fourth, people deemed ineligible because they were unemployed or were not representative of covariates considered in the study (failure to answer the questionnaires or lack of applicability) were also excluded (*n* = 8138). Finally, the analyzed sample comprised 5813 participants (men: 2821 and women: 2992).

### Variables

Dyslipidemia, the dependent variable in this study, was diagnosed based on the levels of total, high density lipoprotein (HDL), low-density lipoprotein (LDL) cholesterol, and triglycerides in blood samples collected after 9–12 h of fasting. According to the 2015 Korean Guidelines for the Management of Dyslipidemia, one of the following four criteria was required: (a) total cholesterol ≥240 mg/dL, (b) HDL cholesterol ≤40 mg/dL, (c) LDL cholesterol ≥160 mg/dL, or (d) triglycerides ≥200 mg/dL [11].

The main independent variable was the work pattern, which included three categories: day, night, and other shifts. The day shift was defined as between 6 A.M. and 6 P.M., while the night shift merged both evening (6 P.M.–12 A.M.) and overnight work (12 A.M.–8 A.M.). Other shifts included various types of working patterns, such as alternating shifts (e.g., day-night-day), 24-h shifts (a full 24-h shift followed by a day(s) off), and split shifts (≥2 shifts within a day).

Socio-demographic, economic, health-related, and nutritional factors were also assessed. Socio-demographic factors included age (30–39, 40–49, 50–59, and ≥ 60 years), region (metropolitan or rural), educational level (high school or less or college and/or beyond), and marital status (married or unmarried). Economic factors included the household income (low, mid-low, mid-high, or high) and occupational category (white-, pink-, or blue-collar employment). Health-related factors included eating habits (regular consumption of breakfast, lunch, and dinner or skipping meals), physical activity/week (active: ≥150 min of moderate activity, ≥ 75 min of vigorous activity, or a mixture of both for ≥150 min; inactive: < 150 min of moderate activity, < 75 min of vigorous activity, or a mixture of both for < 150 min), sleep duration (0–6 or ≥ 7 h per night), smoking status (current smoker, ex-smoker, or non-smoker), alcohol consumption status (≥2 times/month or never), body mass index (BMI) defined obesity status (in reference to the Korean guidelines for overweight and obesity; underweight/normal: < 23, overweight: 23–24.9, and obese: ≥25) [17], hypertension (in reference to the Korean guideline for normal BP, < 120/80 mmHg; normal: 90–199 mmHg systolic or 60–79 mmHg diastolic; prehypertension: 120–139 mmHg systolic or 80–89 mmHg diastolic; hypertension: ≥140 mmHg systolic or ≥ 90 mmHg diastolic) [18], and menopausal status (yes or no). Nutritional factors included macronutrient intake (total kcal, protein, fat, and carbohydrate). For the continuous variables (macronutrient intakes), the OR was calculated for every 100-kcal increase in calorie intake and every 10-g increase in protein, fat, and carbohydrate intake.

### Statistical analysis

All statistical analyses were performed using SAS version 9.4 (SAS Inc., Cary, NC, USA). The chi-square (χ [2]) test was used to evaluate the general characteristics of the study population. For continuous variables (macronutrient intake), a t-test was used to calculate the means and standard deviations. A multiple logistic regression analysis was used to calculate the odds ratios (ORs) with 95% confidence intervals (CIs) in three different models. Model 1 yielded a crude OR, model 2 was adjusted for socio-demographic and economic factors, and model 3 was adjusted for all socio-demographic, economic, health-related, and nutritional factors. Multiple logistic regression analyses of subgroups were also performed to examine the association between night work and dyslipidemia according to occupational category, eating habits, and sleep duration. A general linear model analysis was also used to calculate the mean levels of the four diagnostic determinants (total cholesterol, HDL cholesterol, LDL cholesterol, and triglycerides), and the distributions and percentages of each were calculated. The stratified, clustering, and weight variables developed by the KNHANES were applied to all analyses to improve the representativeness of the sample and account for the limited proportion of participants retained in the final analysis [19]. The significance level was set at *p* value < 0.05.

## Results

Table 1 summarizes the general characteristics of the study population, which included 2821 men and 2992 women. A total of 816 (28.9%) men and 469 (15.7%) women had dyslipidemia. Of the 196 male participants who reported working at night, 76 (38.8%) had dyslipidemia, and the prevalence of dyslipidemia was greater among these night workers compared to those who worked at other times (day: 684/2404, 28.5%; other shifts: 56/221, 25.3%). A similar trend was observed among the female participants, as 70 of the 379 women (18.5%) who reported working at night had dyslipidemia (day: 382/2512, 15.2%; other shifts: 17/101, 16.8%).

**Table 1:** General characteristics of the study population

Variables	Dyslipidemia													
	Male									Female				
	TOTAL		Yes		No		*P-value*	TOTAL		Yes		No		*P-value*
	N	%	N	%	N	%		N	%	N	%	**N**	**%**	
Work pattern							0.004							0.252
Day	2404	85.2	684	28.5	1720	71.5		2512	84.0	382	15.2	2130	84.8	
Night	196	6.9	76	38.8	120	61.2		379	12.7	70	18.5	309	81.5	
Other shifts	221	7.8	56	25.3	165	74.7		101	3.4	17	16.8	84	83.2	
Age(years)							0.011							<.0001
30~39	741	26.3	210	28.3	531	71.7		830	27.7	85	10.2	745	89.8	
40~49	738	26.2	236	32.0	502	68.0		997	33.3	119	11.9	878	88.1	
50~59	634	22.5	196	30.9	438	69.1		742	24.8	163	22.0	579	78.0	
≥ 60	708	25.1	174	24.6	534	75.4		423	14.1	102	24.1	321	75.9	
Region							0.586							0.314
Metropolitan	1696	60.1	497	29.3	1199	70.7		1861	62.2	282	15.2	1579	84.8	
Rural	1125	39.9	319	28.4	806	71.6		1131	37.8	187	16.5	944	83.5	
Educational level							0.348							<.0001
≤ Highschool	1541	54.6	457	29.7	1084	70.3		1812	60.6	343	18.9	1469	81.1	
≥ College	1280	45.4	359	28.0	921	72.0		1180	39.4	126	10.7	1054	89.3	
Occupational categoriesª							0.156							<.0001
White	1085	38.5	336	31.0	749	69.0		1275	42.6	144	11.3	1131	88.7	
Pink	355	12.6	101	28.5	254	71.5		859	28.7	157	18.3	702	81.7	
Blue	1381	49.0	379	27.4	1002	72.6		858	28.7	168	19.6	690	80.4	
Household income							0.179							<.0001
Low	272	9.6	79	29.0	193	71.0		295	9.9	75	25.4	220	74.6	
Mid-low	679	24.1	184	27.1	495	72.9		701	23.4	115	16.4	586	83.6	
Mid-high	910	32.3	251	27.6	659	72.4		908	30.3	121	13.3	787	86.7	
High	960	34.0	302	31.5	658	68.5		1088	36.4	158	14.5	930	85.5	
Marital status							0.898							0.087
Living w/ spouse	2465	87.4	712	28.9	1753	71.1		2384	79.7	360	15.1	2024	84.9	
Living w/o spouse	356	12.6	104	29.2	252	70.8		608	20.3	109	17.9	499	82.1	
Eating habit(daily)							0.014							0.564
Regularly eat breakfast, lunch, and dinner	1772	62.8	484	27.3	1288	72.7		1625	54.3	249	15.3	1376	84.7	
Skip meal(s)	1049	37.2	332	31.6	717	68.4		1367	45.7	220	16.1	1147	83.9	
Physical activity							0.356							0.145
Active	1549	54.9	437	28.2	1112	71.8		1528	51.1	254	16.6	1274	83.4	
Inactive	1272	45.1	379	29.8	893	70.2		1464	48.9	215	14.7	1249	85.3	
Sleep duration(hours)							0.077							0.833
0~6	1460	51.8	401	27.5	1059	72.5		1570	52.5	244	15.5	1326	84.5	
≥ 7	1361	48.2	415	30.5	946	69.5		1422	47.5	225	15.8	1197	84.2	
Smoking status							<.0001							0.346
Current smoker	1046	37.1	352	33.7	694	66.3		135	4.5	17	12.6	118	87.4	
Ex-smoker	1149	40.7	310	27.0	839	73.0		131	4.4	25	19.1	106	80.9	
Non-smoker	626	22.2	154	24.6	472	75.4		2726	91.1	427	15.7	2299	84.3	
Drinking status							0.097							0.001
≥ 2 times / month	2063	73.1	579	28.1	1484	71.9		1420	47.5	189	13.3	1231	86.7	
Never	758	26.9	237	31.3	521	68.7		1572	52.5	280	17.8	1292	82.2	
BMI^b^							<.0001							< 0.0001
Obese(≥25)	809	28.7	257	31.8	552	68.2		564	18.9	129	22.9	435	77.1	
Overweight(23~24.9)	855	30.3	291	34.0	564	66.0		667	22.3	138	20.7	529	79.3	
Normal+underweight(< 23)	1157	41.0	268	23.2	889	76.8		1761	58.9	202	11.5	1559	88.5	
Hypertension							0.102							< 0.0001
Hypertension	627	22.2	160	25.5	467	74.5		375	12.5	82	21.9	293	78.1	
Pre-hypertension	897	31.8	269	30.0	628	70.0		601	20.1	111	18.5	490	81.5	
Normal	1297	46.0	387	29.8	910	70.2		2016	67.4	276	13.7	1740	86.3	
Diabetes							0.006							0.102
Diabetes mellitus	176	6.2	49	27.8	127	72.2		78	2.6	19	24.4	59	75.6	
Impaired fasting glucose	641	22.7	154	24.0	487	76.0		400	13.4	62	15.5	338	84.5	
Normal	2004	71.0	613	30.6	1391	69.4		2514	84.0	388	15.4	2126	84.6	
Menopause														< 0.0001
Yes								834	27.9	193	23.1	641	76.9	
No								2158	72.1	276	12.8	1882	87.2	
Year							0.604							0.014
2013	747	26.5	216	28.9	531	71.1		764	25.5	119	15.6	645	84.4	
2014	704	25.0	200	28.4	504	71.6		722	24.1	90	12.5	632	87.5	
2015	668	23.7	206	30.8	462	69.2		689	23.0	129	18.7	560	81.3	
2016	702	24.9	194	27.6	508	72.4		817	27.3	131	16.0	686	84.0	
Calorie intake(Kcal)^c^	2468.3	±1007.7	2466.7	±1106.9	2468.9	±964.3	0.958	1806.7	±712.2	1803.5	±753.0	1807.3	±704.2	0.914
Protein intake(g)^c^	86.3	±60.3	88.3	±87.4	85.5	±44.8	0.259	62.8	±31.8	62.0	±30.8	62.9	±32.0	0.567
Fat intake(g)^c^	53.1	±40.6	52.8	±46.5	53.2	±37.9	0.788	40.4	±28.6	37.2	±28.1	40.9	±28.7	0.010
Carbohydrate intake(g)^c^	361.9	±132.9	358.9	±128.3	363.2	±134.8	0.439	286.8	±115.9	294.0	±128.4	285.4	±113.4	0.141
Total	2821	100.0	816	28.9	2005	71.1		2992	100.0	469	15.7	2523	84.3	

*BMI* body mass index

^a^Three groups based on International Standard Classification Occupations codes

^b^Obesity status defined by BMI based on 2014 Clinical Practice Guidelines for Overweight and Obesity in Korea

^c^Mean and Standard deviation (SD) of the continuous independent variables in this study

Table 2 summarizes the results from the multiple logistic analysis of the association between night work and dyslipidemia. In all three models, the association between night work and dyslipidemia remained statistically significant in male participants (model 1: OR = 1.58, 95% CI: 1.12–2.21; model 2: OR = 1.61, 95% CI: 1.13–2.29; model 3: OR = 1.53, 95% CI: 1.05–2.24). By contrast, however, no statistically significant association of night work with dyslipidemia was observed in female participants. However, women aged 50 years or older were more likely to have dyslipidemia, compared to their younger counterparts (50–59 years: OR = 1.61, 95% CI: 1.04–2.50; ≥60 years: OR = 1.66, 95% CI: 0.92–3.01).

**Table 2:** Odds ratio for dyslipidemia

Variables	Dyslipidemia																							
	Model 1								Model 2								Model 3							
	Male				Female				Male				Female				Male				Female			
	OR	95% CI			OR	95% CI			OR	95% CI			OR	95% CI			OR	95% CI			OR	95% CI		
Work pattern																								
Day	1.00				1.00				1.00				1.00				1.00				1.00			
Night	1.58	(1.12	–	2.21)*	1.16	(0.82	–	1.64)	1.61	(1.13	–	2.29)*	1.19	(0.82	–	1.72)	1.53	(1.05	–	2.24)*	1.12	(0.76	–	1.66)
Other shifts	0.78	(0.55	–	1.11)	0.90	(0.50	–	1.62)	0.84	(0.58	–	1.21)	0.95	(0.53	–	1.72)	0.84	(0.58	–	1.21)	0.94	(0.50	–	1.74)
Age(years)																								
30~39									1.00				1.00				1.00				1.00			
40~49									1.20	(0.93	–	1.56)	1.22	(0.85	–	1.74)	1.34	(1.03	–	1.75)*	1.19	(0.82	–	1.73)
50~59									1.07	(0.80	–	1.42)	1.98	(1.39	–	2.81)*	1.31	(0.96	–	1.77)	1.61	(1.04	–	2.50)*
≥ 60									0.82	(0.60	–	1.13)	2.02	(1.29	–	3.16)*	1.13	(0.79	–	1.62)	1.66	(0.92	–	3.01)
Region																								
Metropolitan									1.00				1.00				1.00				1.00			
Rural									1.00	(0.83	–	1.22)	1.07	(0.83	–	1.37)	1.03	(0.84	–	1.26)	1.08	(0.83	–	1.39)
Educational level																								
≤ Highschool									1.39	(1.09	–	1.77)*	1.19	(0.82	–	1.73)	1.34	(1.04	–	1.71)*	1.15	(0.78	–	1.69)
≥ College									1.00				1.00				1.00				1.00			
Occupational categoriesª																								
White									1.00				1.00				1.00				1.00			
Pink									0.73	(0.53	–	1.01)	1.29	(0.88	–	1.90)	0.68	(0.49	–	0.96)	1.24	(0.84	–	1.83)
Blue									0.78	(0.60	–	1.03)	1.33	(0.89	–	1.99)	0.76	(0.58	–	1.01)	1.27	(0.83	–	1.95)
Household income																								
Low									0.93	(0.64	–	1.37)	1.35	(0.86	–	2.12)	0.93	(0.63	–	1.38)	1.33	(0.82	–	2.14)
Mid-low									0.75	(0.57	–	0.98)	0.90	(0.66	–	1.24)	0.72	(0.55	–	0.95)	0.87	(0.63	–	1.21)
Mid-high									0.82	(0.65	–	1.04)	0.79	(0.59	–	1.06)	0.81	(0.64	–	1.03)	0.76	(0.57	–	1.03)
High									1.00				1.00				1.00				1.00			
Marital status																								
Living w/ spouse									1.00				1.00				1.00				1.00			
Living w/o spouse									1.05	(0.79	–	1.40)	1.19	(0.90	–	1.56)	1.05	(0.79	–	1.41)	1.14	(0.87	–	1.51)
Eating habit(daily)																								
Regularly eat breakfast, lunch, and dinner																	1.00				1.00			
Skip meal(s)																	1.19	(0.96	–	1.47)	1.42	(1.09	–	1.85)*
Physical activity																								
Active																	1.00				1.00			
Inactive																	0.99	(0.82	–	1.20)	0.82	(0.65	–	1.03)
Sleep duration(hours)																								
0~6																	0.84	(0.69	–	1.02)	0.83	(0.66	–	1.05)
≥ 7 ≥ 7																	1.00				1.00			
Smoking status																								
Current smoker																	1.70	(1.29	–	2.24)*	0.87	(0.45	–	1.67)
Ex-smoker																	1.24	(0.95	–	1.61)	1.55	(0.95	–	2.54)
Non-smoker																	1.00				1.00			
Drinking status																								
≥ 2 times / month																	0.82	(0.66	–	1.01)	0.79	(0.62	–	1.00)
Never																	1.00				1.00			
BMI^b^																								
Obese(≥25)																	1.74	(1.36	–	2.23)*	1.92	(1.43	–	2.58)*
Overweight(23~24.9)																	1.86	(1.48	–	2.33)*	1.67	(1.24	–	2.26)*
Normal+underweight(< 23)																	1.00				1.00			
Hypertension																								
Hypertension																	0.79	(0.60	–	1.03)	1.00	(0.68	–	1.45)
Pre-hypertension																	0.91	(0.74	–	1.12)	1.08	(0.79	–	1.48)
Normal																	1.00				1.00			
Diabetes																								
Diabetes mellitus																	0.93	(0.59	–	1.44)	1.39	(0.73	–	2.66)
Impaired fasting glucose																	0.65	(0.51	–	0.83)	0.80	(0.57	–	1.15)
Normal																	1.00				1.00			
Menopause																								
Yes																					1.43	(0.99	–	2.08)
No																					1.00			
Year																								
2013																	1.04	(0.79	–	1.37)	1.06	(0.71	–	1.59)
2014																	1.01	(0.77	–	1.33)	0.65	(0.45	–	0.94)
2015																	1.15	(0.86	–	1.54)	1.09	(0.78	–	1.53)
2016																	1.00				1.00			
Calorie intake(Kcal)^c^																	1.00	(0.98	–	1.03)	1.03	(0.94	–	1.12)
Protein intake(g)^d^																	1.03	(0.99	–	1.05)	1.00	(0.94	–	1.07)
Fat intake(g)^d^																	0.96	(0.92	–	1.00)	0.89	(0.82	–	0.98)
Carbohydrate intake(g)^d^																	1.00	(0.99	–	1.01)	1.00	(0.97	–	1.04)

Model 1: unadjusted; Model 2: adjusted for age, region, educational level, occupational categories, household income, marital status; Model 3: adjusted for age, region, educational level, occupational categories, household income, marital status, eating habit, physical activity, sleep duration, smoking status, drinking status, BMI, hypertension, diabetes, menopause, micronutrients, and year

*BMI* body mass index

^a^Three groups based on International Standard Classification Occupations codes

^b^Obesity status defined by BMI based on 2014 Clinical Practice Guidelines for Overweight and Obesity in Korea

^c^Per 100 (Kcal) increase

^d^Per 10 (g) increase

*P < 0.05

Table 3 summarizes the results from subgroup analyses stratified by occupational categories, eating habits, and sleep duration. Male night workers who reported skipping meals were more likely to have dyslipidemia, compared to their day working counterparts (OR = 1.63, 95% CI: 1.00–2.67). Similarly, male night workers who slept for 0–6 h were more likely to have dyslipidemia, compared to their day working counterparts. Among female participants, a strong significant association was observed between the occupational category and dyslipidemia, as female night workers with white collar jobs had a nearly three-fold risk of dyslipidemia, compared to their day working counterparts (OR = 2.95, 95% CI: 1.68–5.16).

**Table 3:** The results of subgroup analysis of dyslipidemia to work pattern stratified by occupational categories, eating habit, and sleep duration

Variables	Dyslipidemia								
	Day	Night				Other shifts			
	OR^a^	OR^a^	95% CI			OR^a^	95% CI		
Male									
Occupational categories^b^									
White	1.00	1.75	(0.95	–	3.24)	1.56	(0.60	–	4.04)
Pink	1.00	1.14	(0.50	–	2.61)	0.86	(0.39	–	1.89)
Blue	1.00	1.70	(0.99	–	2.94)	0.72	(0.44	–	1.18)
Eating habit(daily)									
Regularly eat breakfast, lunch, and dinner	1.00	1.56	(0.85	–	2.86)	0.72	(0.44	–	1.16)
Skip meal(s)	1.00	1.63	(1.00	–	2.67)*	1.16	(0.64	–	2.10)
Sleep duration(hours)									
0~6	1.00	1.75	(1.04	–	2.93)*	0.91	(0.54	–	1.54)
≥ 7 ≥ 7	1.00	1.34	(0.78	–	2.31)	0.79	(0.46	–	1.36)
Female									
Occupational categories^b^									
White	1.00	2.95	(1.68	–	5.16)*	0.23	(0.03	–	1.76)
Pink	1.00	0.85	(0.49	–	1.45)	1.65	(0.67	–	4.09)
Blue	1.00	0.48	(0.20	–	1.14)	1.06	(0.37	–	2.99)
Eating habit(daily)									
Regularly eat breakfast, lunch, and dinner	1.00	1.76	(0.99	–	3.01)	0.68	(0.21	–	2.18)
Skip meal(s)	1.00	0.80	(0.49	–	1.30)	1.01	(0.46	–	2.22)
Sleep duration(hours)									
0~6	1.00	1.28	(0.77	–	2.12)	1.00	(0.50	–	2.04)
≥ 7	1.00	1.03	(0.60	–	1.75)	0.88	(0.28	–	2.74)

^a^OR adjusted for all sociodemographic, economic, health-related, and nutritional factors considered in the study

^b^Three groups based on International Standard Classification Occupations codes

*P < 0.05

Table 4 individually summarizes the mean values of the four dyslipidemia diagnostic parameters: (a) total cholesterol, (b) HDL cholesterol, LDL, (c) cholesterol, and (d) triglycerides, as well as the related distributions and percentages of the study sample. Among male subjects, night workers were generally more likely to present with dyslipidemia, compared to their counterparts with other work shift patterns, with 8.2, 16.3, 3.6, and 15.3% meeting the respective criteria of ≥240 mg/dL total cholesterol, ≤40 mg/dL HDL cholesterol, ≥160 mg/dL LDL cholesterol, and ≥ 200 mg/dL triglycerides.

**Table 4:** Mean values of total cholesterol, HDL cholesterol, LDL cholesterol, and triglycerides

Variable	Dyslipidemia												
		Yes^a^						No^a^					
	Total	N	%	Mean ± SD			*P-value*	N	%	Mean ± SD			*P-value*
Male (*n* = 2821)													
Total cholesterol		≥240 ≥ 240 ≥ 240					0.044	< 240 < 240 < 240					0.475
Day	2404	170	7.1	254.852941	±	14.6		2234	92.9	185.707623	±	27.5	
Night	196	16	8.2	247.125	±	5.4		180	91.8	183.117978	±	29.3	
Other shifts	221	10	4.5	247.9	±	12.4		211	95.5	185.149038	±	26.8	
HDL cholesterol		≤40 ≤ 40 ≤ 40					0.532	> 40					0.455
Day	2404	346	14.4	34.8849827	±	3.5		2058	85.6	52.1910633	±	9.8	
Night	196	32	16.3	35.6163125	±	3.5		164	83.7	51.7899506	±	9.9	
Other shifts	221	31	14.0	34.9723226	±	3.4		190	86.0	51.3105455	±	9.0	
LDL cholesterol		≥160					0.049	< 160					0.904
Day	2404	71	3.0	175.915493	±	14.4		2333	97.0	112.69986	±	24.7	
Night	196	7	3.6	168.428571	±	8.6		189	96.4	114.042254	±	27.0	
Other shifts	221	5	2.3	162	±	3.9		216	97.7	112.460317	±	22.8	
Triglycerides		≥200					0.552	< 200					0.765
Day	2404	285	11.9	296.74386	±	142.1		2119	88.1	104.792435	±	38.6	
Night	196	30	15.3	310.733333	±	167.1		166	84.7	106.871951	±	37.8	
Other shifts	221	21	9.5	267.47619	±	72.4		200	90.5	105.822335	±	39.6	
Female (*n* = 2992)													
Total cholesterol		≥240 ≥ 240					0.288	< 240 < 240					0.481
Day	2512	190	7.6	260.110526	±	17.5		2322	92.4	185.358222	±	26.7	
Night	379	33	8.7	256.0	±	12.6		346	91.3	183.956268	±	28.3	
Other shifts	101	10	9.9	254.7	±	14.2		91	90.1	182.835165	±	30.4	
HDL cholesterol		≤40					0.848	> 40					0.003
Day	2512	140	5.6	35.6	±	3.5		2372	94.4	57.6918124	±	11.1	
Night	379	21	5.5	35.6	±	3.7		358	94.5	59.1713183	±	11.8	
Other shifts	101	6	5.9	36.5	±	2.5		95	94.1	60.7348632	±	11.2	
LDL cholesterol		≥160					0.746	< 160					0.551
Day	2512	56	2.2	174.303571	±	13.0		2456	97.8	110.192771	±	25.0	
Night	379	8	2.1	177.5	±	14.8		371	97.9	110.836735	±	24.1	
Other shifts	101	4	4.0	172.0	±	7.4		97	96.0	104.608696	±	31.2	
Triglycerides		≥200					0.871	< 200					0.009
Day	2512	82	3.3	269.743902	±	88.5		2430	96.7	87.3859794	±	36.0	
Night	379	24	6.3	280.0	±	84.5		355	93.7	85.0284091	±	36.9	
Other shifts	101	2	2.0	262.5	±	70.0		99	98.0	76.5555556	±	33.9	

*HDL* high density lipoprotein*, LDL* low density lipoprotein

^a^Cut-offs according to the 2015 Korean Guidelines for the Management of Dylipidemia

## Discussion

After controlling for socio-demographic, economic, health-related, and nutritional factors, we found that night work increased the risk of dyslipidemia in the male participants. Physiological activities, such as eating patterns, lipid/carbohydrate/glucose metabolism, and sleep, all operate on day/night rhythms [20] that are controlled by the circadian biological clock [20]. Accordingly, work schedules that extend beyond the standard 9 A.M.–5 P.M. period impair the circadian rhythm [21]. Night work-related disruptions of the biological clock are likely to result in obesity, impaired insulin secretion, and aberrant glucose homeostasis [20, 22]. Notably, overlap has been observed between the mechanisms associated with insulin resistance and atherosclerosis (a consequence of dyslipidemia), including elevated levels of glucose and free acids that cause oxidant stress, the activation of proinflammatory pathways, low levels of HDL, and high levels of triglycerides [23, 24]. The circadian clock is a key regulator of lipid metabolism and therefore, the lipid profile [25, 26], and periodic disruption of circadian rhythm negatively affects lipid metabolism [26, 27]. Accordingly, night work is more strongly associated with dyslipidemia, compared to day or other shift work.

Meal skipping is a common practice in modern society. Commonly, constant changes in the daily routines of night workers lead to irregular meal times. In our subgroup analysis, we observed a significant positive association of night work with dyslipidemia among male participants who reported skipping meals. Several previous studies reported that these workers tend to skip meals and snack more frequently during the night shift [28–30]. Additionally, compared with regular eaters, meal skippers have higher average values of mean weight, BMI, and triglycerides, which have all been identified as risk factors for dyslipidemia [31].

.Sleep deprivation negatively affects metabolism and impairs the homeostatic control of energy intake (i.e., protein, fat, and carbohydrate) [28, 32], while also promoting the development of an atherogenic lipid profile [33]. These effects explain the significant association between sleep duration and dyslipidemia in this study. Specifically, night workers who slept for < 7 h per night faced a higher risk of dyslipidemia, compared to their counterparts who reported more sleep. The National Sleep Foundation recommends that adults sleep for 7 h per night [34]. According to previous studies, permanent night workers receive less sleep than day workers [35, 36]. Night workers who sleep during the day will inevitably be exposed to light, which hinders the duration and quality of sleep [37]. Specifically, light is the main environmental regulator of circadian rhythm. As the human brain tends to wake when the environment transitions from darkness to light [38], night workers find it difficult to sleep during the day.

Previous studies have reported higher rates of physical inactivity and obesity among white-collar workers, particularly female workers, than those in other occupations [39, 40]. Furthermore, physical inactivity during working hours negatively affects the health of white-collar workers [41]. Both obesity and physical inactivity have been recognized as risk factors for dyslipidemia. These findings seem relevant to our findings, as our subgroup analysis showed a significant association between night work and dyslipidemia among female white-collar workers. Notably, age also correlated directly with the prevalence of dyslipidemia in women, particularly among menopausal women older than 50 years of age. This may be attributable to lipoprotein changes associated with menopause [42], which include increased levels of total and LDL cholesterol [42, 43].

This study had several limitations. First, the cross-sectional design rendered us unable to determine a causal relationship between night work and dyslipidemia. Second, the durations of day, night, and other shift work could not be determined because of limitations of the KNHANES questionnaire. Finally, the key covariates considered in this study, including the sleep duration and eating habits, were self-reported and may have been subject to recall bias. Despite these limitations, this study also featured strengths. This study involved a large, well-validated dataset collected from a nationally representative sample of the South Korean population. Therefore, the findings will likely support the development of interventions and health policies aimed at the increasing problem of dyslipidemia in this population. The study thus makes a relevant contribution to the fields of cardiovascular medicine and epidemiology. Additionally, the KNHANES questionnaires are updated annually to incorporate changes in the real-life health circumstances of South Koreans. Therefore, KNHANES data have been used widely in health-related studies and have provided meaningful insights to inform health policy development in South Korea.

## Conclusions

The findings of previous studies suggest an association of an irregular work schedule, particularly nighttime work, with an altered lipid profile. Accordingly, in this study, we examine the association between night work and dyslipidemia in a nationally representative sample of South Korean adults aged ≥30 years who participated in the KNHANES 2013–2016. In the overall analysis, we found a significant association of night work with dyslipidemia only among male workers. Additionally, subgroup analyses of male workers who reported skipping meals or receiving < 7 h of sleep per night revealed associations of night work with dyslipidemia. Among female participants, a subgroup analysis of white-collar workers found that those who worked at night faced higher risk of dyslipidemia, compared to their day working counterparts.

However, our study was unable to determine a causal relationship between the onset of dyslipidemia and night work, and further investigations are needed to validate the findings of our study. Given the increasing prevalence of dyslipidemia in South Korea and the association of this condition with cardiovascular disease, we also suggest the development of future interventions intended to alleviate dyslipidemia among night workers and ease the burden of CVD in South Korea.

## Abbreviations

CVD: Cardiovascular disease
HDL: High-density lipoprotein
KNHANES: Korea National Health and Nutrition Examination Survey
LDL: Low-density lipoprotein
